# Severe Traumatic Brain Injuries and Associated Outcomes at a Level 1 Trauma Center

**DOI:** 10.3390/biomedicines13071614

**Published:** 2025-07-01

**Authors:** Bharti Sharma, Tirth Patel, Hasan Al-Ali, George Agriantonis, Navin D. Bhatia, Carrie Garcia, Praise Nesamony, Jasmine Dave, Juan Mestre, Shalini Arora, Saad Bhatti, Zahra Shafaee, Suganda Phalakornkul, Kate Twelker, Jennifer Whittington

**Affiliations:** 1 Department of Surgery, NYC Health and Hospitals, Elmhurst, NY 11373, USA; tirth.patel@icahn.mssm.edu (T.P.); agriantg@nychhc.org (G.A.); bhatian1@nychhc.org (N.D.B.); carrie.garcia@nychhc.org (C.G.); nesamonp@nychhc.org (P.N.); davej@nychhc.org (J.D.); mestreju@nychhc.org (J.M.); arorash@nychhc.org (S.A.); bhattisa@nychhc.org (S.B.); shafaeez1@nychhc.org (Z.S.); phalakos@nychhc.org (S.P.); harrisj20@nychhc.org (J.W.); 2 Department of Surgery, Icahn School of Medicine at Mount Sinai, New York, NY 10029, USA; 3 Department of Medicine, St. George’s University School of Medicine, Grenada FZ818, West Indies; halali@sgu.edu

**Keywords:** trauma, brain injury, severity, length of stay, emergency department, mechanisms, mortality

## Abstract

**Background:** Severe traumatic brain injury (TBI) remains a leading cause of mortality and long-term morbidity, particularly in high-acuity trauma settings. We aim to evaluate the clinical, physiologic, and socioeconomic factors associated with outcomes in patients with severe traumatic brain injury (TBI) at a single urban Level 1 trauma center. **Method:** This is a single-center, retrospective study of patients presenting with severe TBI between 1 January 2020 and 31 December 2023 at Elmhurst Hospital Center in Queens, New York. Patients were identified using ICD trauma codes and an Abbreviated Injury Severity (AIS) Head score of ≥3. Demographic data, injury characteristics, vital signs, airway interventions, alcohol level, and insurance status were analyzed. **Result:** A total of 1130 patients met the inclusion criteria. The cohort was predominantly male (76.1%) with a mean age of 52.7 years. Blunt trauma accounted for 97.8% of cases, with a mortality rate of 13.8%, while penetrating trauma comprised 2.2%, with a markedly higher mortality rate of 48%. Patients who died as full code had lower mean systolic blood pressure (82.5 mmHg), oxygen saturation (63%), and shorter emergency department stays (~3.7 h). The mean Glasgow Coma Scale (GCS) score was 12.6, dropping to 6.0 in patients who died. Moreover, higher AIS Head and Injury Severity Score (ISS) values were correlated with worse outcomes. Severely intoxicated patients had higher TBI incidence, with no clear difference observed when compared to normal BAC levels. Self-pay patients exhibited the highest mortality (40%). All associations were statistically significant (*p* < 0.0001). **Conclusions:** Severe TBI outcomes are significantly influenced by injury mechanisms, physiologic parameters, and socioeconomic status. These findings emphasize the need for targeted prognostic tools and improved trauma system preparedness for TBI patients at risk of poor outcomes.

## 1. Introduction

Traumatic brain injury (TBI) remains one of the leading causes of death and long-term morbidity worldwide, particularly in populations requiring high-acuity trauma care [1]. It is defined as a disturbance of brain function caused by an external mechanical force, leading to a spectrum of cranium-implicated pathologies [2]. Classification of TBI severity ranges from mild to moderate to severe, where a severe TBI, typically characterized by a Glasgow Coma Scale (GCS) score of ≤8, has the highest burden of morbidity and mortality [3]. Patients with severe TBI often require intensive care and prolonged hospitalization, leading to long-term rehabilitation with substantial implications for both individual outcomes and healthcare systems [4].

In the setting of trauma, classifying the dynamics of injury can help differentiate certain aspects of injury. TBI can be classified as either penetrating or non-penetrating (blunt) [5]. Penetrating injuries involve direct disruption of the cranial vault and underlying brain structures, most commonly due to gunshot wounds or sharp objects [6]. In contrast, blunt TBIs can be caused by acceleration–deceleration forces such as motor vehicle collisions, falls, and assaults [6]. The mechanism of injury is essential in understanding the physical insult, predicting clinical trajectory, and determining appropriate surgical or non-operative interventions. Studies suggest that penetrating injuries are often associated with higher early mortality, while blunt injuries may involve diffuse axonal injuries and lead to prolonged rehabilitation [6,7].

Furthermore, assessing injury severity is critical for prognostication and clinical planning, necessitating the standardized scales to group injury severity. The GCS is widely used in the acute setting to evaluate neurologic function and guide early management [3]. The Abbreviated Injury Scale (AIS) provides an anatomical severity score for individual body regions, with a head AIS of ≥3 indicating serious injury [8]. Collectively, the Injury Severity Score (ISS), calculated from the three most severely injured body regions based on AIS, reflects the overall trauma burden [9]. These scoring systems are fundamental tools used in trauma registries and research to standardize severity comparisons, guide triage, and predict outcomes across institutions, particularly level 1 trauma centers.

Throughout the advancement of medicine, outcomes following severe TBI vary widely, making it vital to understand the outcome correlations to improve patient care in trauma centers further. Outcomes range from routine home discharge to inpatient rehabilitation, and may also include death, either despite full-code measures or following withdrawal of life-sustaining therapy [10]. Additional variables such as airway management strategies, blood alcohol content (BAC) at presentation, and payer type are potential factors influencing outcomes. These variables may contribute to worsening clinical status in TBI patients and can further aid in predicting possible outcomes.

This study aims to investigate the associations and correlations between injury severity (GCS, AIS, ISS), mechanisms of injury (blunt vs. penetrating), patient-level variables (vitals, airway interventions, BAC), and socioeconomic status (payer type) with clinical outcomes in patients presenting with TBI at a Level 1 trauma center. Identifying these correlations is essential for improving outcome anticipation in critical care fields and the role of factors in clinical outcomes.

## 2. Methods

### 2.1. Overview

This is a single-center, retrospective review conducted at a level 1 trauma center verified by the American College of Surgeons in Queens, New York City. We included all patients who presented with a severe traumatic brain injury between 1 January 2020 and 31 December 2023, inclusive. Patient data were requested from the National Trauma Registry of the American College of Surgeons (NTRACS) Database at our center (Elmhurst Hospital Center). Patients were identified based on the injury mechanism, cause of injury, primary mechanisms (lCD9 or lCDL0 E-Code), and the Abbreviated Injury Severity (AIS) score on the head. The AIS score ranges from 1 to 6 per body region. This study received approval from the institutional review board (IRB) at Elmhurst Facility with IRB number 24-12-092-05G.

### 2.2. Data Collection and Cleaning

We included all patients who presented with a severe TBI between 1 January 2020 and 31 December 2023, inclusive. All patients with an Abbreviated Injury Severity (AIS) ≥3 were included. We excluded patients with AIS less than 3 (minor and moderate injuries). We found a total of 1130 patients with severe TBIs, and all were included in the study. In this included patient population, there were no missing data points. We utilized two sources to obtain complete information on the patient population. First, we requested protected health information (PHI) and other relevant data elements from NTRACS, our center’s trauma registry. If the registry did not include a specific data point for any patient, we used PHI, such as the medical record number (MRN) and the patient’s name, to review their chart and obtain the missing information [11,12,13,14].

We collected data using a data collection tool (Excel sheet or spreadsheet). We incorporated all data elements into this tool. Examples of data elements are demographics (for example, age, sex, race, and ethnicity), AIS, traumatic brain injury pattern, discharge disposition, mortality status, and others. The dataset underwent several preprocessing steps to ensure data integrity, confidentiality, and suitability for statistical analysis. First, to de-identify the dataset, unique identifiers such as medical record numbers (MRNs), dates of birth (DOBs), and patient names were removed. This process was performed to maintain patient privacy in compliance with ethical research standards. Next, inclusion and exclusion criteria were followed, as discussed above, followed by data analysis [11,12,13,14].

### 2.3. Statistical Analysis

Descriptive statistics were utilized to summarize the study cohort. Categorical variables were analyzed using Pearson’s chi-square test or Fisher’s exact test, as appropriate. Measures of effect size, including Cramer’s V, Phi coefficient, and contingency coefficient, were calculated to assess the strength of associations. All statistical analyses were conducted using web-based SAS Studio OnDemand for Academics v9 (version # v3.8).

## 3. Results

A total of 1130 patients with severe traumatic brain injury (TBI) were included in the analysis. According to Table 1, the mean age was 52.67 years (SD ± 41.89), the mean weight was 87.19 kg, and the mean height was 165.43 cm. Most of the cohort were male (76.1%, n = 860), while females comprised 23.9% (n = 270). Males were younger on average (48.96 years) than females (64.50 years), and they were also taller and heavier, with mean heights of 168.50 cm and 156.06 cm and weights of 91.08 kg and 75.18 kg, respectively. When analyzed by race, Black patients (n = 89) had the highest average weight (103.36 kg) and height (172.50 cm), while Asian patients (n = 171) had the lowest average weight (73.27 kg) and were shortest (162.06 cm). White patients (n = 201) had intermediate measures with an average weight of 93.94 kg and height of 169.80 cm. Ethnically, Hispanic patients (n = 521, 46%) were younger (mean age = 46.83 years), while non-Hispanic patients (n ≈ 563, 50%) were older (mean age = 58.60 years). Occupational analysis was not applicable in the majority. All cases were from the state of New York, with patients most commonly presenting from Queens.

Data were collected on outcomes in patients with different types of TBI and associated presenting factors such as fall height, systolic and diastolic blood pressures (BP), heart rate, oxygen saturation (O_2_), respiratory rate, and body temperature. Blunt trauma was the most common mechanism of injury, accounting for 97.8% of cases (n = 1103), while penetrating trauma accounted for 2.2% (n = 25). Among the 97.8% of blunt trauma, 52.2% were discharged to home/self-care, 6.4% died as full code, and 3.1% died following withdrawal of care. Other common discharge destinations included home with services (5.3%), inpatient rehabilitation (5.1%), traumatic brain rehabilitation (4.3%), and skilled nursing facilities (3.0%). In contrast, amongst the 2.2% of penetrating trauma, only 0.6% were discharged to home/self-care, and 0.8% died as full code. Considering that brain death is part of the mortality spectrum, using Table 2, we can infer mortality rate comparison amongst both mechanisms of injury, assuming mortality includes the following as a numerator: died unknown, died after withdrawal of care, died as full code, died with care not begun (DNR/DNI), and met brain death criteria. Using those values divided by total injuries for each type, we can infer that the mortality rate in the blunt TBI category was 13.8%; meanwhile, penetrating TBI had a 48% mortality rate. Fall height was available for patients with blunt trauma and averaged 3.5 m (SD ± 7.5). The highest average fall height was observed among patients who died as full code (15.3 m). Vital signs varied significantly across outcome groups. Among patients with blunt trauma, the mean systolic blood pressure (SBP) was 134.5 mmHg (SD ± 33.8), but patients who died as full code had a mean SBP of only 82.5 mmHg. Similarly, mean diastolic blood pressure (DBP) was 81.5 mmHg but dropped to 52.8 mmHg among deceased as full code patients. On the other hand, penetrating trauma patients had a mean SBP of 117.7 mmHg (SD ± 62.3) and DBP of 83.7 (SD ± 47.3) mmHg. Heart rate followed similar trends in both categories, with blunt trauma patients averaging 86 bpm, but dead at full code patients averaging just 57 bpm. Oxygen saturation was markedly reduced in patients who died (~63%) compared to the overall mean of ~95%, and body temperature was also lower in patients with fatal outcomes. Respiratory rate was generally stable at around 18 breaths per minute across all groups.

Concerning patient disposition of all TBIs, data were collected on associated lengths of stay, ventilation periods, and severity scores across different hospital departments. Emergency department length of stay (EDLOS) averaged 13.1 h (SD ± 13.7) but was significantly shorter for patients who died as full code (~3.7 h), while longer stays were observed in those discharged to rehabilitation areas, such as subacute inpatient rehabilitation (~14.2 h). Hospital length of stay (HLOS) was 11.8 days on average (SD ± 21.1), with the shortest stays in routine discharges (~7 days) and the longest in patients receiving traumatic brain rehabilitation (~36.4 days). Intensive care unit (ICU) stay averaged 3.8 days (SD ± 7.3), with longer durations for patients in rehabilitation (~11.7 days). Ventilation duration followed a similar trend: average 1.7 days overall, peaking at patients ending in skilled nursing facilities (12.4 days), death following withdrawal of care (8.1 days), traumatic brain rehabilitation (5.3 days), and deceased patients (1.9 days), whilst patients who were routinely discharged had a mean duration of 0.4 days. Severity scores showed a clear relationship with outcomes of TBIs. The mean Injury Severity Score (ISS) was 18.7 (SD ± 10.4), with routine discharges having an average of 15.2 (SD ± 6.1), but among patients who died as full code, ISS was significantly higher at 35.9 (SD ± 18.1). Glasgow Coma Scale (GCS) scores had a cohort average of 12.6 (SD ± 4.1), while patients who died as full code had a mean GCS of 6.0 (SD ± 4.8), and routine discharges had a mean of 14.2 (SD ± 2.2). Lastly, the average AIS Head score was 3.7 (SD ± 0.8), with full code deaths having an average of 4.3 (SD ± 1.0) and routine discharges being 3.5 (SD ± 0.7).

The status of patients presenting to the hospital was also collected. Of the 29 patients who arrived without signs of life, all (100%) were declared dead. Among the 1,099 who arrived with signs of life, the majority (54.2%) were discharged, and only 12.2% were declared dead.

Airway management data of presenting TBIs showed that 220 patients (19.5%) underwent oral endotracheal intubation, while 890 (78.9%) had no documented airway intervention. Among intubated patients, mortality was significantly higher: 25.5% died as full code and 10% after withdrawal of care, with total mortality being 45%. In contrast, routine discharge was the most common outcome for non-intubated patients (61.8%), with mortality being 6.3%.

TBI trauma volume varied across the calendar year, with the highest case counts occurring in September (n = 130, 11.5%), followed by July (n = 115, 10.2%) and August (n = 104, 9.2%). The lowest number of cases occurred in April (n = 68, 6%). Mortality was highest in September and December (11 full-code deaths each) and lowest in April (1 death). Routine discharges were most frequent in September, in line with the overall seasonal trend.

In addition to Table 2 for trauma type, trauma injury additionally shows that the cause of injury further influenced outcomes: falls, assaults, and motorcycle collisions (MCC) were associated with better outcomes, where discharges occurred in 50.7% of falls, 67.2% of assaults, and 64.0% of MCC. Additionally, mortality was 11.9% in falls, 5.7% in assaults, and 14.4% in MCCs. On the other hand, gunshot wound (GSW) TBIs had no routine discharges and a mortality rate of 72.7%. (7.3%).

The alcohol level at presentation (ETOH class) shown in Table 3 shows that severe toxic intoxication is associated with a higher incidence of TBI when compared to other levels of toxicity, and additionally, 32 mortalities, compared to 5 and 7 in mild and moderate, respectively. However, severe intoxication presented a mortality ratio of 10.5%, compared to 13.8%, 27.8%, and 10.1% seen in patients with normal, mild, and moderate blood alcohol levels, respectively. Furthermore, the discharge ratio was 67.3% in severe blood alcohol levels, compared to 57.4%, 55.6%, and 53.6% in normal, mild, and moderate levels, respectively.

Statistical tests confirmed significant associations: trauma type (χ^2^ = 49.47, *p* < 0.0001), cause of injury (χ^2^ = 522.96, *p* < 0.0001), and alcohol level (χ^2^ = 215.08, *p* < 0.0001) in Table 3a,b, Table 4 and Table 5, respectively. Standard Chi-Square test showed statistically significant findings across trauma type, cause, and ethanol classification, with a *p*-value less than 0.0001. Likelihood ratio (LR) showed a significant association between variables and outcome by comparing the observed data in a fitted model. After adjustment for confounding, trauma cause showed a significant Mantel–Haenszel chi-square analysis, indicating no confounding variables could have influenced the cause and outcome relation. This was not observed in trauma type and ethanol classification after adjustment. All findings were supported in Table 6 by Fisher’s exact test, showing a value less than 0.0001. Effect sizes showed weak associations for all three (Cramer’s V ranging from 0.1759 to 0.2183). However, Phi and contingency coefficients showed stronger associations in trauma cause and ethanol classification.

Insurance status on Table 7 was also significantly correlated with disposition. Medicaid covered the largest proportion of patients (n = 428, 37.9%), with a mortality rate of 9.3% and discharge rate of 67.5%. Medicare patients (n = 273, 24.2%) had the highest mortality rate (16.1%) and a lower discharge rate (48.4%). Commercial insurance patients (n = 142, 12.6%) showed a mortality rate of 10.6% and a discharge rate of 64.8%. The self-pay group (n = 30, 2.7%) had the most concerning profile, with a mortality rate of 40%. These differences were statistically significant (χ^2^ = 130.79, *p* < 0.0001) in Table 8 across the chi-squared test and its likelihood ratio, and significance was confirmed via Fisher’s exact test in Table 9. Mantel–Haenszel showed that confounding variables cannot be ruled out based on their value. Effect size analysis showed a weak to moderate association (Phi = 0.3402; contingency coefficient = 0.3221; Cramer’s V = 0.1964). Monte Carlo on Table 10 Estimate provides a confidence interval for the exact test (to 0.0005).

## 4. Discussion

### 4.1. Outline of Study

This study offers a comprehensive evaluation of the clinical, demographic, and socioeconomic factors with associated outcomes in patients with severe traumatic brain injury (TBI) at a Level 1 trauma center. The findings confirm several previously established patterns and provide new insights regarding outcome variability associations in this critical patient population. The use of initial presenting signs, trauma mechanisms, standardized scales, airway interventions, alcohol intoxication status, and payer type as predictors of outcome degree provides a multifactorial view that can inform management and future interventions.

### 4.2. Demographics and Seasonal Insight

Consistent with the trends observed in our cohort, male predominance in severe TBI presentations remains a striking and consistent feature. This gender disparity, with males comprising over three-quarters of the sampled population, highlights potential biological, behavioral, and societal factors that predispose younger males to high-risk situations, leading to head trauma. As suggested from previous studies, this patient group could have higher risk-taking behaviors, participation in high-velocity activities, and occupational hazards, which may collectively contribute to this representation [15]. These findings suggest a need for targeted injury prevention strategies focusing on younger male populations, specifically through public health interventions aimed at education and behavioral modification.

Age-related ethnic differences were also notable, with Hispanic patients presenting at a younger mean age compared to non-Hispanic patients. In parallel, variations in biometric profiles, where black patients demonstrated higher average weight and height than other racial groups, raise important considerations regarding injury biomechanics. Heavier and taller individuals may experience a different force distribution during trauma. In contrast, the shorter and lighter body habitus observed among the Asian patients may correspond differently, which can account for the higher incidence of these patients in this cohort. These differences are not fully elucidated within the scope of this study; however, they merit further biomechanical investigation to better understand their clinical relevance in TBI prevalence.

Seasonal variation in TBI incidence is also observed, with a consistent trend showing an increased case volume noted in late summer and early fall, peaking in September, and a decreased incidence in April. Mortality rate followed the stated trend, peaking in September. Such seasonal dynamics highlight the importance of considering temporal factors in trauma system preparedness. Trauma centers may benefit from preventive strategies seasonally to anticipate and mitigate the increased burden observed during those high-risk months.

### 4.3. Initial Injury Mechanism and Associated Factors

Injury mechanism profoundly influenced outcomes. Penetrating TBI accounted for a small proportion (2.2%) but was associated with disproportionately high mortality (48%), compared to blunt trauma, which had a 13.8% mortality rate. These findings, not only being statistically significant in our study, but they also align with previous studies, which demonstrated that penetrating brain injuries, especially gunshot wounds as indicated in our study, confer significantly higher mortality relative to blunt trauma [7]. This disproportionate mortality emphasizes the fundamentally distinct pathophysiological consequences of penetrating trauma. Penetrating injuries often involve direct laceration or disruption of critical brain structures, major vascular injuries, and high intracranial contamination risks, all of which contribute to rapid clinical deterioration and limited therapeutic options. In contrast, blunt trauma typically induces secondary brain injuries such as diffuse axonal injury, cerebral edema, and hematomas, which may offer a broader window for medical and surgical intervention. An additional notable finding was the higher count of blunt TBI patients requiring extended care with rehabilitation services, with outcomes compared to penetrating TBIs, including inpatient rehabilitation (58 vs. 1), skilled nursing facilities (34 vs. 3), and traumatic brain rehabilitation (49 vs. 2). This pattern reflects the nature of blunt trauma, which often results in diffuse and multifocal brain injuries that, while not immediately fatal, lead to substantial long-term neurological impairment. The extended course of care needed for functional recovery emphasizes the chronic impact of severe blunt TBI. These findings highlight the importance of early rehabilitation planning and resource allocation for blunt trauma patients, even when initial survival is achieved.

Our study shows that vital signs at presentation are strong clues of outcome. Hypotension and hypoxia were markedly associated with higher mortality, supporting prior evidence that secondary brain injury due to systemic hypotension and hypoxia can independently worsen outcomes [16]. Deceased patients had dramatically lower mean systolic blood pressures (~82.5 mmHg) and oxygen saturations (~63%) than survivors at presentation. Additionally, hypothermia was noted in patients who had outcomes of mortality. In contrast, the respiratory rate failed to provide differences in either outcome. These signs demonstrated significant prognostic value in predicting outcomes following severe TBI. Such evidence reinforces the critical role of early physiological stabilization, particularly aggressive management of hypotension and hypoxia, in influencing survival. Prompt and effective resuscitation strategies in the prehospital and early hospital phases may represent one of the few modifiable factors capable of altering the trajectory of outcomes of severe TBI.

Our findings of blunt TBI fall height analysis showed that greater heights correlated with increased mortality, especially falls reaching 15 m, seen in patients with resultant death despite full code measures. On the other hand, shorter fall height was observed in patients with outcomes of discharge and rehabilitation measures, indicating a linear association of fall height vs. outcome. This suggests that a higher fall height would exacerbate the severity of TBI, or in other words, the increase in distance of fall would yield higher velocity of impact, which can amplify the blunt injury, causing poorer outcomes with a higher fall, and more likely favorable outcomes with a shorter fall. However, fall injuries are often underestimated, particularly in elderly populations, due to risk factors in select patient populations, where those of age extremes can have more devastating impacts independent of fall height. This is further stated in a study, which states that ground-level falls in the elderly population possess higher long-term mortality rates than other causes [17]. Therefore, this association cannot apply to asserted populations, as other unidentified factors affect injury severity.

With regards to the cause of injury, as expected, penetrating injuries such as gunshot wounds (GSW) were linked to the worst prognosis, with GSW-related TBI carrying an alarming 72.7% mortality rate and no routine discharges. This was primarily the most notable association with increased mortality rate, as this type of injury would be under the criteria of a penetrating TBI. Statistical significance was also noted in this variable for outcome, with Mantel–Haenszel analysis showing that no confounding variable could have affected these results, further strengthening its association. Patients with GSW have linked association to decreased survival rate possible due to the extensive injury associated with cranial fracture, brain tissue laceration from impact and impulse forces of the bullet, and increased risk of multi-vascular injury of blood supply; all of which can result is rapid loss of viable tissue, limiting treatment options for survivability. However, aggressive early management is still beneficial to gunshot wound TBI patients, as evidenced by past papers, which additionally claims the trajectory of GSW is crucial in distinguishing the severity of such injury, where trauma being trans-ventricular or bi-hemispheric leads to poorer outcomes [18]. This contrasts with lower mortality injuries such as falls, assaults, and motorcycle collisions, which had higher incidence but better discharge and rehabilitation ratios. This finding suggests that the stated injuries are likely blunt TBIs, explaining the pattern of outcomes.

Alcohol intoxication at presentation was an interesting finding; blood alcohol levels were classified into normal, mild, moderate, and severe levels. As expected, higher TBI prevalence was observed in patients with severe alcohol levels when compared to other toxicity levels, if present. However, a unique finding was a small difference in mortality and discharge ratios amongst patients who were severely intoxicated and those with normal BAC. This finding, although associated with higher TBI incidence, suggests no increased mortality and morbidity with severe toxicity in those patients when compared to individuals who presented with moderate to no alcohol levels. Interestingly, alcohol has been theorized to have a protective effect on TBI injuries as evidenced by animal models [19]; however, many publications still consistently indicate severe intoxication is significantly associated with increased rate of in-hospital complications and mortality [20]. Regardless, our findings reported statistical significance that BAC affects outcome, with slightly lower mortality rates in the severely intoxicated cohort; other papers have documented findings that suggest an insignificant effect of alcohol on TBI severity and lengths of stay [21]. On the other hand, due to its increased TBI incidence, as evidenced by our results, achievements of such levels increase the risk of injury, possibly due to the altered mental status of severely intoxicated patients. Further studies should investigate the association of high blood alcohol content with neurovascular impairment due to TBI and explain such unique findings.

### 4.4. Standardized Severity Scales

Severity scoring systems were strong scales that provided insight into outcomes in this cohort of severe TBI patients. The Glasgow Coma Scale (GCS) demonstrated a strong association with mortality. Patients who died as full code had a markedly lower mean GCS of 6.0 (SD ± 4.8), compared to survivors discharged routinely, who exhibited a mean GCS of 14.2 (SD ± 2.2). Given that GCS evaluates core neurologic functions of eye, verbal, and motor response, it remains a rapid yet powerful tool for initial assessment in the trauma setting. Additionally, this line includes claims of studies where a lower GCS score at presentation yielded higher mortality risk [3].

Similarly, the AIS of the head region also proved to be a meaningful predictor of outcome. Patients who faced complications had a higher mean AIS Head score of 4.3 (SD ± 1.0) compared to those who were routinely discharged, who had a lower mean score of 3.5 (SD ± 0.7). These results highlight that even within the severe injury spectrum (≥3), a small increase in the anatomical head injury severity, as captured by AIS Head scoring, has important implications for survival. This ability to quantify isolated head trauma severity is crucial in grading the extent of damage and can be used in acute care settings to predict outcome associations.

Finally, when evaluating the overall burden of injury, the Injury Severity Score (ISS) offered additional insights beyond neurologic status alone. Patients who died as full code exhibited significantly elevated ISS values (mean 35.9, SD ± 18.1) compared to routine discharges (mean 15.2, SD ± 6.1), reflecting the compounded impact of multisystem trauma on outcomes. As ISS aggregates the severity of the three most severely injured body regions, it provides a valuable measure for understanding the cumulative systemic insult beyond the focus of the brain injury. It aids in predicting mortality in the wider scope of patients who are involved in multisystem trauma.

Together, these severity indices—GCS for neurologic function, AIS Head for focal cranial injury severity, and ISS for whole-body trauma assessment—proved to be essential in predicting outcomes of patients presenting with severe TBI at our Level 1 trauma center.

### 4.5. Airway Measures and Length of Stay

The association between airway management and mortality is also studied in our cohort. Intubated patients exhibited a higher mortality rate (45%) compared to non-intubated patients (6.3%). While this may initially suggest an adverse effect of intubation, it more likely reflects the severity of neurological compromise that prompts airway control, necessitating the need for advanced airway management to stabilize the patient. Of the 1128 patients, 890 did not need any form of intubation, suggesting those who underwent such measures were likely to have been in a poorer state initially, affecting their mortality risk. Regardless, prior studies support that intubation is often required in patients with GCS scores ≤8 and significant hypoxia, which are themselves predictors of poor outcome as stated above [22]. From the means of intubation offered in this cohort, endotracheal intubation dominates as the most common form, with 220 patients of the 238 that had airway support measures undergoing endotracheal intubation. The discharge ratio of non-intubated patients was also significantly higher, 61.8%, whilst the discharge ratio of patients who underwent oral endotracheal intubation was much lower, standing at a rate of 18.6%, with higher risks of mortality and rehabilitation courses. Therefore, a patient undergoing such a measure is unlikely to be routinely discharged like a patient who did not get intubated. Such results ask whether the initial patient status that prompted airway maneuvers is responsible for the outcome, or whether those measures have an independent effect.

Length of stay metrics across emergency, inpatient, and intensive care settings also reflected significant variation based on patient outcomes and care trajectories. Patients who ultimately died as full code had the shortest emergency department length of stay (EDLOS) (mean ~3.7 h), a finding that likely reflects limited opportunity for stabilization in these critically injured individuals. In contrast, patients discharged to rehabilitation pathways had longer EDLOS, such as those transferred to subacute inpatient rehabilitation (~14.2 h), indicating a more prolonged stabilization or evaluation process before disposition. Hospital length of stay (HLOS) further emphasized the transition of long-term care by displaying its disposition times, where routine discharges occurred after a relatively short hospitalization (~7 days), patients requiring traumatic brain rehabilitation stayed markedly longer (~36.4 days), suggesting that survival alone does not indicate recovery but often necessitates extended multidisciplinary care. ICU length of stay showed a similar trend to HLOS, with an average of 3.8 days across the cohort, increasing to 11.7 days for those requiring rehabilitative services. This prolonged ICU duration may reflect the need for ongoing neuromonitoring, anticipation of complications, and complex medical needs in patients with survivable but severe neurologic deficits resulting from the TBI.

Ventilation duration was also stratified by disposition: patients who died after withdrawal of care or who were discharged to skilled nursing or rehabilitation centers had longer periods of mechanical ventilation, with means ranging from 5.3 to 12.4 days; in contrast, patients who died with full code resuscitative efforts had an average of 1.9 days. This can be a result of acute multisystem decompensation in latter patients due to more severe presentations than when compared to patients who died after withdrawal of care. Full code patients likely would not have benefited from ventilation, as their damage was extensive, such that ventilation would not have extended their stay. Patients who died after withdrawal of care, on the other hand, could be more stable patients with singular system dysfunction that put their life at risk, where turning the ventilator off would cause them to expire. Additionally, routinely discharged patients required minimal ventilatory support (~0.4 days), claiming that brief or absent intubation may be seen in less severe neurologic injury and more favorable recovery trajectories.

These findings collectively bring up the resource-intensive nature of TBI recovery in patients who survive but remain functionally impaired, as seen in those partaking in rehabilitation courses. They also highlight the inverse relationship between survivability and hospital burden: those with the most severe, non-survivable injuries die early and have low utilization of resources, while survivors with major neurologic compromise, and possibly less severe presentations, can often experience prolonged hospitalizations, intensive care stays, and long-term ventilation. From a systems perspective, this emphasizes the need for adequate resource allocation when long-term treatment courses of TBIs are inferred.

### 4.6. Socioeconomic Factors

Socioeconomic factors, particularly insurance status, demonstrated a notable influence on patient outcomes following severe TBI. Patients covered by Medicaid represented the largest subgroup and experienced a moderate mortality rate of 9.3%, suggesting access to basic post-acute care but also reflecting the challenges inherent to low-income populations that cause them to have an increased incidence of TBI. Conversely, patients with Medicare coverage had the second highest incidence but exhibited the highest mortality among insured groups at 16.1%, which may reflect the older age and greater burden of comorbidities typically associated with this population, posing them at higher risk of death secondary to their more alarming risk factors. Strikingly, the self-pay group had an alarmingly high mortality rate of 40%, raising serious concerns about disparities in access to timely, comprehensive trauma care. While the reasons behind this elevated mortality may be multifactorial, ranging from delayed presentation and self-pay population sample, it nevertheless shows the vulnerability of uninsured individuals in high-acuity trauma settings. These findings align with the longstanding concern in the trauma literature regarding socioeconomic differences in survival outcomes, where prior studies indicate that uninsured patients had worse outcomes after trauma, and highlight insurance as an independent factor affecting mortality in post-trauma settings [23]. However, generalization should be avoided because it is based on a smaller sample size.

## 5. Strengths

This study holds both clinical and statistical significance in the understanding of outcome determinants of patients with severe traumatic brain injury. The scope of the study narrows down to TBI injury but stays wide in terms of all its associated factors, which strengthens its analytic detail. It proves essential by its large sample analysis of TBI patients from an urban community over a long period, where different injury mechanisms and types are illustrated in detail alongside highly specific outcomes. Furthermore, unlike previous studies, our research is unique in terms of using multiple models at the same time; it reinforces and compares the predictive value of widely used severity indices such as GCS, AIS Head, and ISS in stratifying mortality and disposition outcomes. Collectively, this study demonstrated how multiple factors were also correlated alongside these outcomes, making it a study that identifies early physiologic indicators such as hypotension, hypoxia, and the need for airway intervention. Moreover, patient-specific influences, such as ethanol intake and insurance status, were also noted. This analysis views the different trajectories of TBI presentations and what outcomes can be predicted from them, making this study essential in detecting broader multifactorial associations.

By capturing a large, diverse cohort and analyzing outcomes across a broad set of variables, this study provides actionable data for anticipating outcomes to help inform triage decisions and resource planning strategies, aiding in reducing disparities and improving TBI care delivery.

## 6. Limitations

Several limitations must be acknowledged. Findings in this study are evidently significant but should be subjected to serious readjustments. As a retrospective analysis, inherent biases such as selection bias and unmeasured confounders are present. Data from a single urban trauma center may limit generalizability to rural or non-Level 1 trauma settings. Moreover, these data were collected from the NTRACS, which may not always provide complete information. Additionally, detailed neuroimaging findings, other in-hospital interventions, and long-term functional impairments were not available but would enhance the depth of analysis. Regarding scales, the Injury Severity Scale (ISS) was used in this study, instead of the refined New Injury Severity Scale (NISS), which is the latest to date. TBI incidents were only recorded at hospital presentations, which limits extremes of injury, such as patients who died before reaching the hospital, and patients who had a milder onset in which they did not need further medical care. Socioeconomic factors were significant to an extent; however, associations cannot be made due to differences in sample size and population. Future studies should avoid these limitations by enforcing up-to-date scoring systems and sourcing data from multi-facility centers to minimize any disparities.

## 7. Future Perspectives

It is important to strengthen TBI findings further to minimize the high mortality rate it possesses. Future studies should focus on reinforcing predictive strategies from the leads provided by this study as a guide for pattern recognition. This study aimed to look at the presenting TBI and follow up with its outcome. However, future studies should investigate the various interventions of TBI, which can help optimize early resuscitation of critically injured patients. Socioeconomic care is to be further analyzed beyond insurance status; such factors can include occupational information. Moreover, integrating advanced imaging biomarkers can also be researched to explore the possibility of outcome prediction by those modalities.

## 8. Conclusions

This study highlights the multifactorial determinants of outcomes in patients with severe traumatic brain injury at a Level 1 trauma center. Clinical indicators such as low GCS, high AIS Head and ISS scores, hypotension, hypoxia, and the need for airway intervention were all strongly associated with increased mortality. Blunt trauma, while associated with lower early mortality, often led to prolonged hospitalization and rehabilitation, whereas penetrating trauma, particularly gunshot wounds, carried the highest mortality risk. Socioeconomic variables, although their generalization is limited by sample size relevant to the population, further influenced outcomes, with self-pay patients showing the highest mortality and severely intoxicated patients demonstrating slightly better discharge rates; however, severe alcohol intoxication showed no overall difference in outcome. These findings emphasize the importance of combining clinical severity scores with physiologic and socioeconomic data to inform prognosis, guide early management, and improve trauma care delivery. Future efforts should anticipate predictive models for outcome estimation in severe TBI.

## Figures and Tables

**Table 1:** Demographics of the sampled population and associated biometric data. Counts and age averages were noted. Weight and height averages are noted with standard deviations.

Demographic and Biometric Characteristics	Age (Years)		Weight		Height	
	Count	Mean	Mean	Std Dev	Mean	Std Dev
**Gender**						
**Female**	270	64.50	75.18	37.10	156.06	16.21
**Male**	860	48.96	91.08	42.64	168.50	14.56
**Race**						
**American Indian**	1	22.32	96.00		178.00	
**Asian**	171	61.57	73.27	32.55	162.06	13.58
**Black**	89	56.63	103.36	53.97	172.50	10.47
**Native Hawaiian or Other Pacific Islander**	5	63.57	75.20	44.57	166.00	8.04
**Other**	640	48.18	86.63	40.19	163.94	15.74
**Unknown**	23	44.20	82.50	37.66	161.82	26.36
**White**	201	58.51	93.94	44.69	169.80	17.35
**Ethnicity**						
**Hispanic Origin**	521	46.83	87.45	41.98	163.21	15.10
**Non-Hispanic Origin**	563	58.60	86.58	41.75	168.19	13.60
**Unknown**	46	46.27	92.00	43.41	154.92	35.10
**Occupation**						
**Building and Grounds Cleaning and Maintenance**	1	68.49	63.00		158.00	
**Community and Social Services Occupations**	1	59.03	124.00		162.00	
**Construction and Extraction Occupations**	12	49.94	89.45	28.90	175.18	5.65
**Food Preparation and Serving Related**	6	44.11	87.17	32.98	169.00	3.74
**Healthcare Practitioners and Technical Occupations**	1	32.91	210.00		66.00	
**N/A**	1	47.52	59.00		179.00	
**Not Applicable**	1094	52.78	86.84	41.23	165.53	15.38
**Production Occupations**	1	66.27	84.00		167.00	
**Sales and Related Occupations**	1	68.88	67.00		56.00	
**Transportation and Material Moving Occupations**	5	46.40	140.80	125.72	168.33	1.53
**Unknown**	7	48.89	84.33	35.44	160.00	10.05
**County**						
**Bronx**	1	33.92	77.00		167.00	
**Kings (Brooklyn)**	72	46.32	86.04	43.50	167.55	20.93
**New York (Manhattan)**	2	59.71	81.00	32.53	175.00	7.07
**Queens**	1055	53.11	87.29	41.85	165.27	15.54
**State**						
**NY**	1130	52.67	87.19	41.89	165.43	15.91
**Total**	1130	52.67	87.19	41.89	165.43	15.91

**Table 2:** Counts of outcomes with associated trauma type.

Table of Disposition by Type			
Disposition	Type		
	Blunt	Penetrating	Total
**Died Unknown**	1	0	1
**Died after withdrawal of care**	35	0	35
**Died as full code**	72	9	81
**Died with care, not begun DNR/DNI**	21	1	22
**Discharged to Home or Self-care (Routine Discharge)**	589	7	596
**Home with services**	60	0	60
**Homeless/Shelter**	8	0	8
**Inpatient Psych Care**	6	0	6
**Inpatient Rehabilitation**	58	1	59
**Left AMA**	31	0	31
**Met brain death criteria**	23	2	25
**New placement at Skilled Nursing Facility**	34	3	37
**Other Acute Care Hospital Emergency Department**	16	0	16
**Other Acute Care Hospital Inpatient**	16	0	16
**Other Health Care Facility**	14	0	14
**Police Custody/Jail/Prison**	5	0	5
**Return to Skilled Nursing Facility**	3	0	3
**Sub-Acute Inpatient Rehabilitation**	62	0	62
**Traumatic Brain Rehabilitation**	49	2	51
**Total**	1103	25	1128
**Frequency Missing = 5**			

**Table 3:** (**a**). Counts of patients disposed of based on groups and their ethanol classification. (**b**). Statistical analysis for a table of disposition by type.

(**a**)						
**Table of Disposition Grouped by ETOH Class**						
**Disposition Group**	**ETOH Class**					
	**Mild**	**Missing**	**Moderate**	**Normal**	**Severe**	**Total**
**Deceased**	5	39	7	81	32	164
**Discharged**	10	74	37	337	206	664
**Missing**	0	0	3	1	1	5
**Other**	3	40	22	168	67	300
**Total**	18	153	69	587	306	1133
(**b**)						
**Statistic**			**DF**	**Value**	**Prob**	
**Chi-Square**			18	49.4749	<0.0001	
**Likelihood Ratio Chi-Square**			18	37.3478	0.0047	
**Mantel–Haenszel Chi-Square**			1	0.2794	0.5971	
**Phi Coefficient**				0.2094		
**Contingency Coefficient**				0.2050		
**Cramer’s V**				0.2094		

**Table 4:** Statistical analysis for a table of disposition by cause.

Statistic	DF	Value	Prob
**Chi-Square**	270	522.9558	<0.0001
**Likelihood Ratio Chi-Square**	270	356.0400	0.0003
**Mantel–Haenszel Chi-Square**	1	6.4916	0.0108
**Phi Coefficient**		0.6812	
**Contingency Coefficient**		0.5630	
**Cramer’s V**		0.1759	

**Table 5:** Statistical analysis for a table of disposition grouped by ethanol classification.

Statistic	DF	Value	Prob
**Chi-Square**	72	215.0783	<0.0001
**Likelihood Ratio Chi-Square**	72	194.2561	<0.0001
**Mantel–Haenszel Chi-Square**	1	0.3722	0.5418
**Phi Coefficient**		0.4367	
**Contingency Coefficient**		0.4002	
**Cramer’s V**		0.2183	

**Table 6:** Statistics for Fisher’s exact test of TBI type, cause, and ethanol classification.

Fisher’s Exact Test	
**Table Probability (*p*)**	<0.0001

**Table 7:** Patient count based on payor group type and their association disposition group.

Table of Payor by Disposition Group					
Payor	Disposition Group				
	Deceased	Discharged	Missing	Other	Total
	13	6	0	2	21
**Auto**	1	7	0	6	14
**Blue Cross**	9	20	0	13	42
**Commercial**	15	92	0	35	142
**Corrections**	0	0	0	1	1
**HMO**	1	4	0	3	8
**Managed Care**	1	1	0	1	3
**Medicaid**	40	289	1	98	428
**Medicaid Pend**	0	2	0	1	3
**Medicare**	44	132	0	97	273
**No Fault**	8	22	0	14	44
**None**	15	41	0	12	68
**Other Govmt**	0	2	0	4	6
**Other**	5	25	0	9	39
**Self Pay**	12	16	1	1	30
**Workers Comp**	0	5	0	3	8
**Total**	164	664	2	300	1130
**Frequency Missing = 3**					

**Table 8:** Statistical analysis for a table of payor group and disposition group.

Statistic	DF	Value	Prob
**Chi-Square**	45	130.7928	<0.0001
**Likelihood Ratio Chi-Square**	45	106.6754	<0.0001
**Mantel–Haenszel Chi-Square**	1	0.0005	0.9829
**Phi Coefficient**		0.3402	
**Contingency Coefficient**		0.3221	
**Cramer’s V**		0.1964	

**Table 9:** Fisher’s exact test of payor type.

Fisher’s Exact Test	
**Table Probability (*p*)**	<0.0001

**Table 10:** Monte Carlo for payor type.

Monte Carlo Estimate for the Exact Test	
**Pr** **≤** **P**	<0.0001
**99% Lower Conf Limit**	<0.0001
**99% Upper Confidence Limit**	0.0005
**Number of Samples**	10000
**Initial Seed**	579,318,266

## Data Availability

The data were requested from the Elmhurst Trauma registry and extracted using electronic medical records after receiving approval from the Institutional Review Board at our facility (Elmhurst Hospital Center).
